# Facilitating ventilator weaning through rib fixation combined with video-assisted thoracoscopic surgery in severe blunt chest injury with acute respiratory failure

**DOI:** 10.1186/s13054-020-2755-4

**Published:** 2020-02-12

**Authors:** Tung-Ho Wu, Hsing-Lin Lin, Yi-Pin Chou, Fong-Dee Huang, Wen-Yen Huang, Yih-Wen Tarng

**Affiliations:** 1 Department of Critical Care Medicine, Kaohsiung-Veterans General Hospital, Kaohsiung, Taiwan; 2 Division of Thoracic Surgery, Department of Surgery, Kaohsiung-Veterans General Hospital, Kaohsiung, Taiwan; 3 Division of Trauma, Department of Emergency, Kaohsiung-Veterans General Hospital, Kaohsiung, Taiwan; 4 Department of Cosmetic Science, College of Human Ecology, Chang Gung University of Science and Technology, Taoyuan, Taiwan; 5 School of Accounting and Finance, Beijing Institute of Technology, Zhuhai, China; 6 Department of Orthopedics, Kaohsiung-Veterans General Hospital, 386, Da-Chung 1st Road, Kaohsiung City, 813 Taiwan

**Keywords:** Ventilator dependence, Rib fixation, Acute respiratory failure, Video-assisted thoracoscopic surgery, Blunt thoracic injury

## Abstract

**Background:**

Severe blunt chest injury sometimes induces acute respiratory failure (ARF), requiring ventilator use. We aimed to evaluate the effect of performing rib fixation with the addition of video-assisted thoracoscopic surgery (VATS) on patients with ARF caused by blunt thoracic injury with ventilator dependence.

**Methods:**

This observational study prospectively enrolled patients with multiple bicortical rib fractures with hemothorax caused by severe blunt chest trauma. All patients received positive pressure mechanical ventilation within 24 h after trauma because of ARF. Some patients who received rib fixation with VATS were enrolled as group 1, and the others who received only VATS were designated as group 2. The length of ventilator use was the primary clinical outcome. Rates of pneumonia and length of hospital stay constituted secondary outcomes.

**Results:**

A total of 61 patients were included in this study. The basic demographic characteristics between the two groups exhibited no statistical differences. All patients received operations within 6 days after trauma. The length of ventilator use was shorter in group 1 (3.19 ± 3.37 days vs. 8.05 ± 8.23, *P* = 0.002). The rate of pneumonia was higher in group 2 (38.1% vs. 75.0%, *P* = 0.005). The length of hospital stay was much shorter in group 1 (17.76 ± 8.38 days vs. 24.13 ± 9.80, *P* = 0.011).

**Conclusion:**

Rib fixation combined with VATS could shorten the length of ventilator use and reduce the pneumonia rate in patients with severe chest blunt injury with ARF. Therefore, this operation could shorten the overall length of hospital stay.

## Introduction

Severe blunt chest trauma can cause multiple rib fractures accompanied by hemothorax or pneumothorax [1, 2] and lung contusions, compromising the facility for gas exchange in the lung parenchyma. Together with severe pain, all these complications can induce acute respiratory failure (ARF)—oximeter saturation of less than 90% and PaO_2_ level of less than 60 mmHg, as determined through a Venturi mask with 100% FiO_2_, such that mechanical ventilation is required—the most serious posttrauma complication, which may induce high mortality.

Intubation with positive pressure ventilation is widely accepted as an initial treatment for ARF [3, 4]. This method can be rapidly applied to provide sufficient tissue oxygenation in patients with lung contusion as well as rib fracture, contributing to impaired gas exchange in the lungs. However, long-term ventilator use should be avoided because ventilator-acquired pneumonia can become another contributor to increased morbidity and length of hospital stay. Therefore, reducing ventilator dependence following major complications is crucial.

Studies have demonstrated that early video-assisted thoracoscopic thoracic surgery (VATS) can decrease the rate of posttrauma pneumonia and empyema in blunt chest trauma [5, 6]. Some studies have claimed that rib fixation can also decrease ventilator dependence [7, 8], but others have considered this claim to be controversial [9, 10]. The results might vary depending on the surgical approach used during rib fixation. In our hospital, we combine VATS and rib fixation in severe blunt chest injuries with ARF. In our previous study, we found that rib fixation exhibited some benefits in patients without respiratory failure [11]. In this study, we hypothesize that rib fixation with VATS could provide a better outcome in intubated patients with ARF. The two study periods overlapped. However, the patient groups were not the same. The main purpose of this study was to evaluate the effects of this approach on the length of ventilator use and length of hospital stay.

## Materials and methods

### Patient set-up

**Fig. 1:**
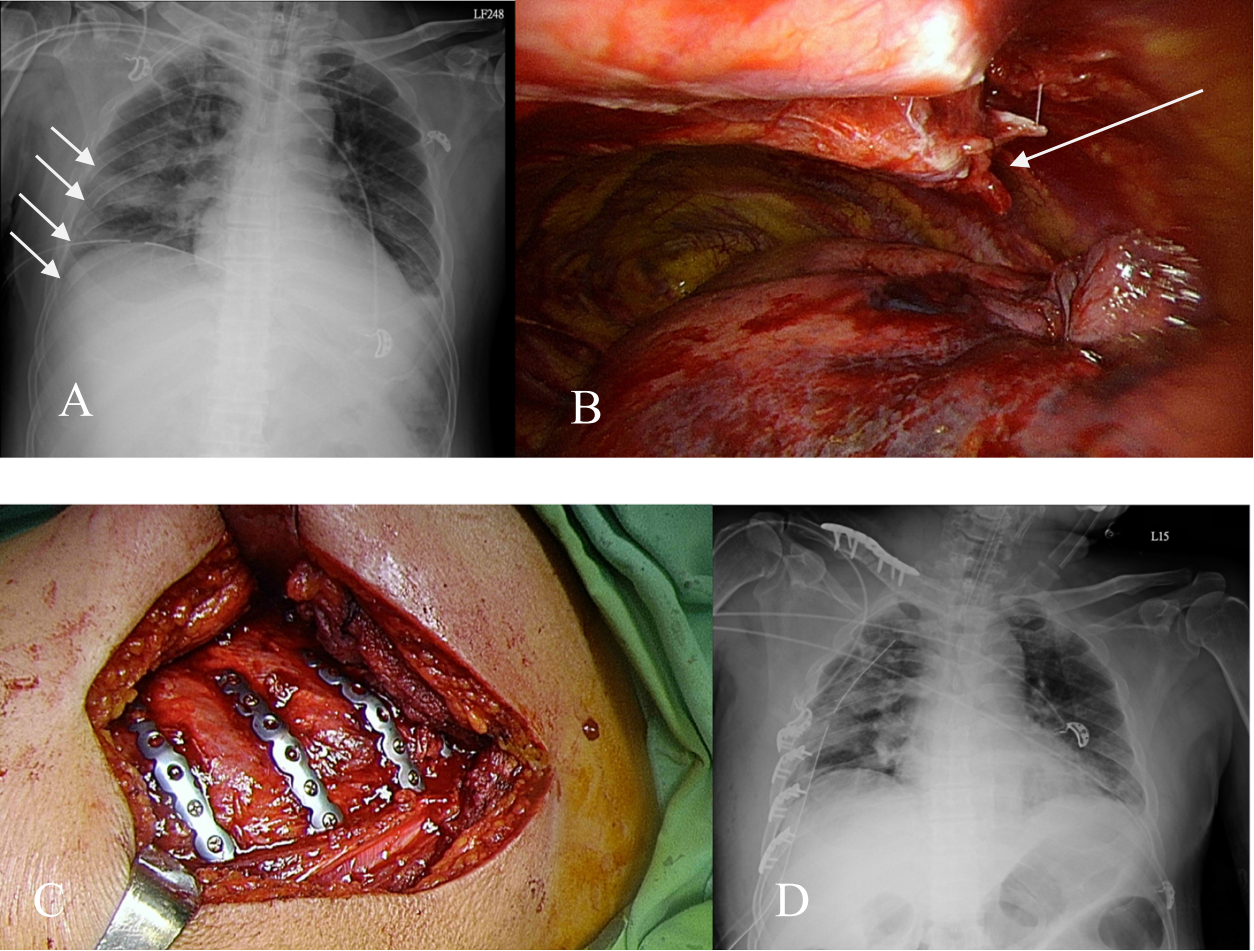
**a** Right side 5th to 8th rib bicortical fractures (white arrow) along with lung contusions. **b** VATS evacuation of hemothorax combined with localization of fractured rib (white arrow). **c** Fixation of fractured ribs using titanium plates with locking screws on the outer surface of ribs. **d** Chest X-ray after the operation

This prospective observational study was established in October 2014. The patient enrollment period was from March 2015 to June 2018 in a level 1 trauma medical center in southern Taiwan that receives approximately 15,000 emergency trauma visits per year. Patients included in this study were 18 years of age or older and had a site of a major blunt chest injury. All of them had ARF within 24 h after trauma. The imaging studies exhibited at least 3 bicortical rib fractures combined with hemothorax, pneumothorax, and lung contusions (Fig. 1a). The abbreviated injury scores (AISs, 2008 edition) for the chest injuries of the patients were 3 or 4 indicated severe injuries. Tube thoracostomies were applied to these patients in the emergency department (ED). Injuries in other regions were evaluated during a secondary survey. The study protocol was approved by our hospital’s ethics committee (VGHKS17-CT12-13).

Patients not receiving intubation with ventilator support were excluded. Patients whose AISs for other regions were higher than 3 or exceeded their chest AISs were excluded to avoid any confounding bias from associated injuries. Patients with bilateral rib fractures were also excluded. Patients with hemodynamic instability or mediastinal great vessel injuries were also excluded. Finally, patients with severe medical diseases, such as liver cirrhosis, end-stage renal disease, chronic obstructive pulmonary disease, or cancer, were excluded as well.

**Fig. 2:**
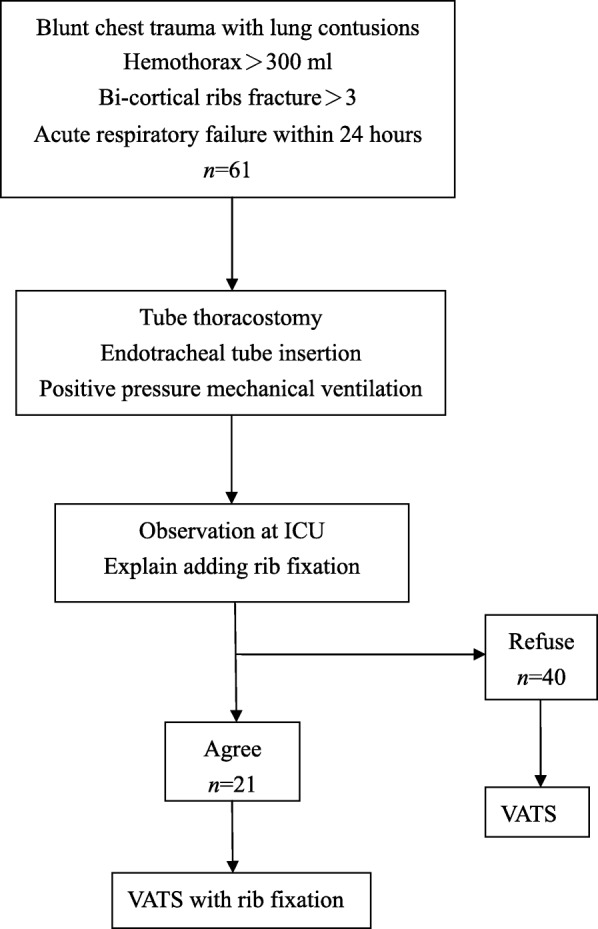
Treatment algorithm for severe blunt chest trauma with acute respiratory failure

All patients received detailed evaluations in the ED, including chest computed tomography. The number of rib fractures, volume of pleural collections, and conditions of lung contusions were realized in detail [12, 13]. After resuscitation, patients were admitted to the intensive care unit (ICU) for further evaluation. In the ICU, ventilators with positive end-expiratory pressure (PEEP) were used for fully sedated patients. Intravenous analgesics were also administered and adjusted by nursing staff according to the critical care pain observation tool. Because all patients were under sedation, patients’ closest relatives were informed of the treatment plans by trauma surgeons. The main treatment goal is to decrease the ventilator-dependent days. VATS for pleural collection and rib fixation for chest wall stability are two primary treatment methods. Patients whose relatives agreed to rib fixation with VATS were enrolled in group 1, and those who only agreed to VATS without rib fixation were included in group 2. These family members provided consent for the procedures. The treatment algorithm is illustrated in Fig. 2.

### VATS and surgical fixation of fractured ribs

All surgical procedures were performed in operating rooms, and patients were under general anesthesia. As per our other studies [14, 15], in the VATS-only group, VATS was applied for the evacuation of residual blood clots and the resection of pulmonary lacerations under endoscopic visions. In patients who received rib fixation, VATS was also first used to treat the pleural and lung parenchyma lesions. The surgical wound incisions were then designed according to the fracture sites of the ribs that were localized using VATS (Fig. 1b). The fractured sites were approached using the chest wall muscle-sparing method [16–18]. Titanium plates with locking screws (MATRIXRIB™, products of DePuy Synthes, companies of Johnson & Johnson) that were specifically designed for fractured ribs were selected and applied at the outer surfaces of the ribs (Fig. 1c). One dose of first-generation cephalosporins as prophylactic antibiotics was administered before the operations.

### Postoperative care

After the operations, all patients underwent chest tube drainage. The chest tubes were connected to a low-pressure suction device (− 15 cm, H_2_O) to reduce residual pleural effusions (Fig. 1d). Ventilators were still applied after operations. A weaning ventilator protocol was planned. When patients achieved optimal oxygen saturation, their endotracheal tubes were removed. Chest tubes were removed when the air leaks were absent, and fluid drainage was less than 100 mL per day. Routine follow-up chest X-rays and close observations for wounds were performed after operations. Residual pleural effusions after operations were managed with secondary VATS. For patients who required ventilator dependence for longer than 14 days, tracheostomies were performed.

### Data collection

The demographic data of all patients were collected, including age, sex, number of fractured ribs, pulmonary contusion score, and trauma mechanism. The AIS of each associated injury was also collected, and the injury severity score (ISS) was calculated. Duration of ventilator use, duration of chest tube use, rates of infection, and total dose of analgesics were recorded. The lengths of stay (LOSs) in the ICU and hospital were recorded as secondary outcomes. If a fever occurred, sputum and blood samples were collected immediately and over the following 3 days for analysis of microbial cultures. In addition, cultures from chest fluid were collected during VATS. The protocol for pain control in the patients with rib fractures was the same as that used in both the study groups for which only morphine was used. Total doses of morphine were also calculated and recorded. All patients’ care followed the same guidelines regarding ventilator settings, and the two groups did not differ during intubation or protocols for extubation.

### Statistical analysis

An initial descriptive analysis was performed on all variables to determine the frequencies and averages in the two groups. Numerical variables are presented as means ± standard deviation (SD). The chi-square and Fisher’s tests were used to evaluate the categorical and proportional variables, respectively, between the two groups. Continuous variables were compared using analysis of variance. We considered *P* < 0.05 to be statistically significant. All data were analyzed using SPSS 20.0 (IBM Corp., Armonk, NY, USA). For this study to achieve a power greater than 0.8 to detect a significant difference in hospital LOS between patients and controls (two-sided equality, two-sample), a sample size of at least 60 patients was required (*α* = 5%, sampling ratio = 0.5). Multivariate logistic regression was used to assess the associated variables of rib fixation. Confounders were controlled using a running entry model of logistic regression analysis. Subsequently, all independent variables were entered into the multivariate logistic regression analysis to detect the independent variables for rib fixation. The Hosmer–Lemeshow test was used to evaluate the goodness of fit. Significant associations of the independent variables with the dependent variables were assessed using a 95% confidence interval (CI) and the respective adjusted odds ratio (aOR).

## Results

During the study period, a total of 61 patients were enrolled. All patients had ARF and received emergent endotracheal tube insertions and positive pressure ventilator support within 24 h after trauma. These patients all had pneumothorax and hemothorax, resulting in tube thoracostomy being performed in the ED. Twenty-one patients receiving rib fixation with VATS were included in group 1, and the other 40 patients receiving VATS only belonged to group 2. The collection was terminated after statistical power reached 0.8.

Table 1 presents the basic demographic characteristics of the two groups. The means of age and the distribution of sex between the two groups had no statistically significant differences. Factors that could affect the ventilator dependence—including consciousness levels, number of fractured ribs, percentage of flail chest, lung contusion scores, AISs, and ISSs—all exhibited no statistically significant differences. All patients were evaluated for pain levels in the ED initially, and the 10-point pain scores between the two groups exhibited no differences. Thus, the two groups were satisfactorily matched and could be compared on the basis of their similar characteristics.

**Table 1:** Comparison of basic demographics between groups

	All patients (*n* = 61)	VATS with rib fixation (group 1; *n* = 21)	VATS only (group 2; *n* = 40)	*P*
Age (mean ± SD)	53.48 ± 18.37	51.29 ± 15.93	54.63 ± 19.62	0.477
Sex (male)	50 (82.0%)	15 (71.4%)	35 (87.5%)	0.121
BMI	25.95 ± 3.97	26.51 ± 3.81	25.65 ± 4.07	0.420
Number of fractured ribs	6.61 ± 2.42	6.57 ± 2.06	6.63 ± 2.61	0.930
Flail chest	26 (42.6%)	10 (47.6%)	16 (40.0%)	0.568
Pulmonary contusion scores	5.70 ± 1.86	6.14 ± 2.48	5.48 ± 1.41	0.263
Time from trauma to VATS	4.49 ± 1.58	4.52 ± 1.69	4.48 ± 1.54	0.913
Lung laceration	17 (27.9%)	6 (28.6%)	11 (27.5%)	0.929
Initial GCS	12.84 ± 3.27	13.57 ± 2.99	12.45 ± 3.38	0.191
Head AIS	1.13 ± 1.18	0.95 ± 0.97	1.23 ± 1.27	0.356
Thoracic AIS	3.54 ± 0.50	3.62 ± 0.50	3.50 ± 0.51	0.383
Abdomen AIS	0.85 ± 1.30	1.10 ± 1.48	0.70 ± 1.20	0.330
Limb AIS	1.70 ± 1.01	1.81 ± 1.03	1.65 ± 1.00	0.565
ISS	21.11 ± 6.69	21.67 ± 6.43	20.83 ± 6.88	0.638
Pain score at ED	8.89 ± 1.36	9.24 ± 1.22	8.67 ± 1.41	0.121

*AIS* abbreviate injury score, *ISS* injury severity score, *SD* standard deviation, *VATS* video-assisted thoracoscopic surgery, *GCS* Glasgow coma scale, *ED* emergency department, *BMI* body mass index

All patients in this study received VATS within 6 days after trauma [19]. The time periods from trauma to operation were equal between the two groups, without statistical significance. In this study, a total of 17 (27.9%) patients received lung repair during VATS. The proportions of patients receiving lung repair in the two groups were similar (Table 1). Lobectomy procedures were not performed in this study. After rib fixations, most patients with ARF could be weaned off the ventilator within 4 days or much faster than VATS-only patients (3.19 ± 3.37 days vs. 8.05 ± 8.23, *P* = 0.002). The total ventilator-dependence time durations were also much shorter in the rib fixation group (7.71 ± 3.65 days vs. 12.55 ± 8.22, *P* = 0.002). A patient was defined as having high PEEP if pressure was greater than 5 cmH_2_O. Patients in group 2 required significantly more days of total ventilator use (5.71 ± 3.65 vs. 10.55 ± 8.22, *P* = 0.013). Eight patients had prolonged ventilations that required a tracheostomy; all these patients belonged to group 2 (0 vs. 20.0%, *P* = 0.042). Because of early weaning from the ventilator in the rib fixation group, the rate of pneumonia was also much lower (38.1% vs. 75.0%, *P* = 0.005). The time of chest tube use after the operation was also shorter in the rib fixation group (6.33 ± 2.13 days vs. 8.75 ± 4.04, *P* = 0.003). Because of early weaning from the ventilator and shortened periods of chest tube use, the LOSs in the ICU and hospital were both shorter in group 1, with statistical significance (8.81 ± 3.40 days vs. 12.08 ± 4.95, *P* = 0.008; 17.76 ± 8.38 days vs. 24.13 ± 9.80, *P* = 0.011). In this study, all patients had normal consciousness upon discharge. The pain scores at discharge were much lower in the rib fixation group (3.57 ± 1.03 vs. 5.18 ± 1.40, *P* = 0.001). Table 2 illustrates all clinical outcomes after operations on patients with ARF.

**Table 2:** Comparison of clinical outcomes between groups

	VATS with rib fixation (group 1; *n* = 21)	VATS only (group 2; *n* = 40)	*P*
Ventilator use after VATS (days)	3.19 ± 3.37	8.05 ± 8.23	0.002
Total ventilator use (days)	5.71 ± 3.65	10.55 ± 8.22	0.013
Rate of tracheostomy	0	7 (17.5%)	0.042
Chest tube use after VATS (days)	6.33 ± 2.13	8.75 ± 4.04	0.003
Total chest tube use (days)	9.33 ± 3.68	12.80 ± 4.55	0.002
Rate of pneumonia	8 (38.1%)	30 (75.0%)	0.005
ICU LOS (days)	8.81 ± 3.40	12.08 ± 4.95	0.008
Hospital LOS (days)	17.76 ± 8.38	24.13 ± 9.80	0.011
Pain score at discharge	3.57 ± 1.03	5.18 ± 1.40	0.001
Morphine dose (mg)	80.24 ± 34.73	124.71 ± 48.88	0.001

*VATS* video-assisted thoracoscopic surgery, *ICU* intensive care unit, *LOS* length of stay

We applied binary logistic regression to test the parameters, and the results are displayed in Table 3. According to the multivariate logistic regression, the only independent clinical outcome determinant of the two groups was the time periods of ventilator use (aOR, 3.411; 95% CI 1.214–9.585, *P* = 0.020). These results indicate a strong association between rib fixation and ventilator dependence. Calibration of the final model was assessed using the Hosmer–Lemeshow goodness of fit test. A *P* value of 0.726 (χ^2^ = 5.290) suggested that the model was accurate.

**Table 3:** Binary logistic regression analysis of associations between the two groups with rib fixation or without rib fixation

Explanatory variables	Odds ratio 95%	Confidence interval	*P* value
Gender	0.014	0.000–0.440	0.015
Age	0.985	0.927–1.045	0.611
Rib fracture numbers	1.016	0.598–1.726	0.955
Lung contusion	0.436	0.223–0.852	0.015
Time of ventilator	3.411	1.214–9.585	0.020
Pneumonia	0.201	0.023–1.725	0.143
Empyema	59.388	0.718–4908.858	0.070
Time from trauma to operation	0.718	0.422–1.224	0.224
GCS	0.739	0.514–1.062	0.102
ISS	0.800	0.452–1.416	0.444
Inhospital LOS	1.145	0.948–1.384	0.161
ICU LOS	0.379	0.133–1.083	0.070

*GCS* Glasgow coma scale, *ISS* injury severity score, *LOS* length of stay

Five patients in this study had residual pleural effusion after the operation. Three patients belonged to group 1, and the other two were from group 2. All these patients successfully received secondary VATS. One patient belonging to group 2 expired during the study period due to acute myocardium infarction. The mortality rate was 1.6% in all patients, without any statistical significance between the two groups (0 vs. 2.5%, *P* = 1).

## Discussion

ARF is a dangerous posttraumatic complication that occurs mainly when both chest wall and lung parenchyma are destroyed in severe blunt chest injuries [20]. This emergency situation requires rapid resuscitation through the immediate insertion of an endotracheal tube with ventilator support. The primary treatment goal for these patients is early weaning from the ventilator. In this study, rib fixation with VATS in patients with such severe blunt chest trauma and ARF could greatly decrease the length of ventilator dependence.

Other studies have usually targeted treatments of severe blunt chest trauma by managing the hemothorax or pneumothorax [21–23]. Initially, fractured ribs were not the main target of treatment in our hospital [23]. In fact, fractured ribs, especially the bicortical fractured type, can produce an unstable thoracic cage that might not support the full expansion of lung parenchyma. Although positive pressure ventilation can provide a supporting force for lung expansion, long-term ventilator dependence may be required to wait for the chest wall to heal. Many orthopedic materials have been designed specifically for fractured ribs. Several studies have successfully proven that reconstruction of a chest wall using rib fixation could reduce ventilator dependence [16, 24, 25]. In contrast with other studies, we performed rib fixation combined with VATS to completely manage chest wall injury, lung laceration, and pleural collections. These two procedures can be performed simultaneously to accelerate ventilator weaning. The rate of ventilator-acquired pneumonia subsequently decreases, which may also shorten the whole course of treatment.

The timing of the operation is another influential factor in blunt chest trauma. Early VATS may decrease the rate of pleural infection. Our previous studies have demonstrated that VATS should be performed within 1 week after trauma [19]. We thus adhered to this principle in this study. All patients received VATS within 6 days after trauma. The rates of empyema for the two groups are similar without statistical significance. Group 1 patients had rib fixation added during VATS to enable the reconstructed chest wall to maintain a firm structure. A stable chest wall can allow patients with lung parenchyma to retain full lung capacity without long-term use of the high PEEP ventilator mode. This benefit could decrease the probability of barotrauma for alveoli. The earlier the expansion of lung parenchyma occurs, the earlier the ventilator weaning can occur.

Fractured ribs not only make the chest wall unstable but also result in the persistence of pleural collection, especially bicortical fractures [26, 27]. The fractured ends of ribs can produce an ooze from the bone marrow [28]. The fractured ribs could also scrub lung surfaces, which may induce persistent microair leakage. These complications both lengthen the usage time of chest tubes, which is another major factor that lengthens the total course of admission. In this study, rib fixation was observed to prevent these complications. Chest tube usage time after rib fixation was much shorter than in the VATS-only group.

In this study, the pain score was difficult to estimate because all patients had endotracheal insertions with sedation during ventilator dependence. Only one pain evaluation was conducted, upon initial arrival at the ED, and all patients had similar pain scores. During ventilator-dependent periods, pain agents were administered continuously. The longer the period was for ventilator dependence, the more analgesics and sedative agents were required. In this study, after patients were weaned from the ventilator, they all regained normal consciousness. The pain scores upon discharge were evaluated again and had decreased compared with initial admission scores. In the rib fixation group, the pain score upon discharge was lower than in the other group. Therefore, pain can be greatly reduced for patients undergoing rib fixation compared with those who do not receive rib fixation.

Although VATS combined with rib fixation has many advantages in patients with ARF, this study has several limitations. The numbers of patients with ARF are small for thoracic injuries because only 5 to 10% experience incidences after blunt chest trauma. However, in this study, we attempted to include a sufficient number of patients for the calculation power to reach 0.8. The other limitation of the study was that patient selection was not randomized, although the study protocol was designed prospectively. Because all patients were initially intubated and sedated after trauma, all decisions regarding operation were made by patients’ families. We considered that all patients in this study should receive fixation but that consent should be obtained from families. Although we provided detailed information regarding rib fixation, some family members did not understand our explanations. Therefore, we could only provide their families with choices to inform their final decisions. However, this condition provided a comparison group for the study. Although the two groups were not matched initially, which may have caused selection bias, the demographic characteristics of the two groups exhibited no statistically significant differences. The binary logistic regression examination also indicated a high correlation between rib fixation and the length of ventilation use after adjustment for other variances. Another limitation of the observation-based data was that different staff members recorded the observations. To mitigate this limitation, a senior surgeon reviewed all data for accuracy and consistency. All surgical procedures were performed by the same team, and the same surgeons were responsible for all decisions to perform rib fixation to avoid surgical confounding bias. The pain score evaluation comparison between the two groups was also a limitation in this study. In addition to the subjectivity of pain sensation, group 2 patients had longer ICU stays that may have interfered with pain scores. Fortunately, all patients in this study were lucid and conscious, decreasing the potential for such bias.

## Conclusion

The combination of rib fixation with VATS may provide complete treatment of chest wall deformity and of lung and pleural lesions in severe blunt chest injuries. This combination surgery may decrease ventilator dependence, lower the rate of pneumonia, and shorten the course of admission.

## Data Availability

The datasets generated and analyzed during the current study are available from the corresponding author on reasonable request (Tung-Ho Wu, Hsing-Lin Lin1, Yi-Pin Chou, Fong-Dee Huang, Wen-Yen Huang, Yih-Wen Tarng).
